# Towards a comprehensive breastfeeding-friendly workplace environment: insight from selected healthcare facilities in the central region of Ghana

**DOI:** 10.1186/s12889-021-11652-5

**Published:** 2021-09-09

**Authors:** Jacqueline Nkrumah, Aaron Asibi Abuosi, Rodney Buadi Nkrumah

**Affiliations:** 1 Faculty of Science Education, Department of Health Administration and Education, University of Education, P.O Box 25, Central Region, Winneba, West African Ghana; 2 Department of Public Administration and Health Services Management, University of Ghana Business School, P. O. Box 75, Greater Accra Region, Accra-Legon, West Africa Ghana; 3 Center for Research on Children and Families, McGill University|3506 University Street, Suite 106, Montreal, QC H3A 2A7 Canada

**Keywords:** Optimal breastfeeding, Frontline health workers, Breastfeeding workplace environment, Work challenges, Coping strategies

## Abstract

**Background:**

In the last three decades, Ghana has championed the objectives of Baby-Friendly Hospital Initiatives to provide pregnant women and nursing mothers with the skills and support systems necessary for attaining optimal breastfeeding. Yet, little is known in literature on how these intervention regimes practically promote breastfeeding-friendly work environment in healthcare facilities and their level of effectiveness. This study explores the extent to which healthcare facilities in Ghana’s Effutu Municipality provide breastfeeding-friendly workplace environment to breastfeeding frontline health workers.

**Methods:**

A descriptive mixed-method approach was employed to collect data from fifty-four participants, comprising healthcare facility representatives and breastfeeding frontline health workers. A self-administered questionnaire with structured responses was administered to frontline health workers, followed by interview guides for representatives of hospital management. Thematic analysis was used to analyze interview responses. Responses to questionnaires were processed with SPSS version 23.0 and presented using frequencies and percentages.

**Results:**

Three main themes emerged, namely, Standpoints on workplace breastfeeding support; Breastfeeding support, and Suggested future directions. Beyond this, six sub-themes emerged, including backings for workplace breastfeeding support; perceived benefits of breastfeeding support; factors of poor breastfeeding workplace support; maternity protection benefits; workplace support gaps, and awareness creation on benefits. Breastfeeding frontline health workers held that their hospitals have no breastfeeding policy (96%), no breastfeeding facility (96%), they do not go to work with baby (96%), but had 12 weeks maternity leave (96%) and worked half-day upon return to work (70%).

**Conclusion:**

Health facilities in the study do not provide a breastfeeding-friendly work environment except for the privileges provided by the Labor Act and conditions of service. Continuous advocacy on breastfeeding workplace support and stakeholder engagement to build consensus on the mix of strategies suitable to cushion breastfeeding frontline health workers is recommended for optimal breastfeeding and improved productivity.

**Supplementary Information:**

The online version contains supplementary material available at 10.1186/s12889-021-11652-5.

## Background

Optimal breastfeeding practice is known to improve infant and child health and enhance cognitive ability in children [1]. In Ghana, breastfeeding practice is universal among mothers and fifty-two (52%) percent of all infants, aged 0–6 months are exclusively breastfed [2]. However, the country’s effort to improve optimal breastfeeding is hampered by several factors including rapid urbanization and its associated formalization of women’s work [3]. Exclusive breastfeeding takes a dip during the third month of infants’ life because women return to work after 3 months of maternity leave [1]. This situation is partly driven by insufficient organizational-level support systems and individual mother’s attitudes and values [4]. While some employee mothers believe that extension of maternity leave would do the magic, others believe that prioritizing home and workplace support systems for breastfeeding should be the approach to protect and promote optimal breastfeeding [5]. Strategies for scaling up optimal breastfeeding practices, therefore, demand appropriate harmonization of home and workplace factors that promote and protect, breastfeeding appropriately.

Ghana’s strategy towards promoting optimal breastfeeding practice begun with the implementation of the Innocenti Declaration of 1990, 5 years after it has been launched. This involved the implementation of a “*Ten Step*” successful breastfeeding code, and a Baby-Friendly Hospital Initiative (BFHI), both aimed at socializing midwives to become aware of information and skills for breastfeeding as well as providing nursing mothers with the skill set to practice optimal breastfeeding. Becoming Breastfeeding Friendly (BBF) assessments conducted in Ghana showed that the country has a moderate breastfeeding scale-up environment [4, 6, 7]. Consequently, the BFHI has been revived and relaunched to achieve its aim. The initiative is perceived by stakeholders as one of the surest ways to protect and promote breastfeeding. Yet, maternal work, particularly paid work is limiting optimal breastfeeding practice in emerging industrial economies such as Ghana, making breastfeeding and childcare support intervention necessary. If BFHI is a vital element of breastfeeding promotion and support in Ghana, then the extension of this initiative to the workplace cannot be overemphasized. Today, most Ghanaian health facilities are accredited as baby-friendly. Yet, there is little empirical evidence on how these protocols and policies translate into a breastfeeding-friendly workplace environment for Breastfeeding Frontline Health Workers (BFHWs). We test this assertion by exploring the extent to which healthcare facilities at the sub-national level (i.e., Effutu Municipality) provide a breastfeeding-friendly workplace environment for BFHWs using a descriptive mixed-method approach. To achieve this aim, we asked three main questions: 1). To what extent do healthcare facilities in the Effutu Municipality provide a breastfeeding-friendly workplace environment for BFHWs? 2). What views do BFHWs in the Effutu Municipality share on breastfeeding workplace support? 3). What coping strategies do BFHWs employ to balance work and breastfeeding challenges in the Effutu Municipality? By BFHWs, we refer to breastfeeding clinical and non-clinical health staff in health facilities.

### Work, breastfeeding, and workplace support in Ghana

Maternal work and breastfeeding incompatibility have received growing interest from infant feeding researchers in Ghana. It is suggested that women in self-employment and paid work experience tension between work and breastfeeding and are unable to practice exclusive breastfeeding. However, work-breastfeeding tension is experienced differently depending on the type of work a mother does. In some cases, the conflict between work and breastfeeding has led some mothers to withdraw from work or spend less time with babies [8, 9]. Also, it has been found that poor recommendations of exclusive breastfeeding from health workers and shorter duration of maternity leave contribute to the decline in exclusive breastfeeding at 6 months [10]. This has led some scholars to explore the availability and effectiveness of breastfeeding support mechanisms in workplaces. For instance, Mensah [11] studied the role of social support in improving breastfeeding and employee commitment and found that breastfeeding mothers who received support from relatives, spouses, and colleagues appeared more satisfied and committed to their jobs compared to those with no or less social support.

Recent studies have also investigated the lived experiences and views of breastfeeding mothers in paid work and tertiary institutions and have identified individual and organizational-level factors that affect optimal breastfeeding practice. Abekah-Nkrumah and colleagues [12], found factors such as lack of knowledge and understanding of exclusive breastfeeding, negative lived experiences of working mothers, and unsupportive organizational environment as adversely affecting exclusive breastfeeding. A study of institutional-level support for breastfeeding among tertiary students also found a stern conflict between breastfeeding and academic work due to lack of breastfeeding support for student mothers. In this study, student mothers used unapproved and unsafe places as breastfeeding facilities [8].

Even though the above studies suggest a general lack of breastfeeding workplace support, other studies also identify maternity leave, peer support, and breastfeeding break as basic workplace breastfeeding support available in tertiary institutions and healthcare facilities that support working mothers to breastfeed [13]. While the above studies provide evidence of inadequate workplace breastfeeding support in Ghana and breastfeeding mothers’ lived experience of work-breastfeeding tensions, they only present workplace breastfeeding support from employees’ perspective and do not bring to bear the views of employers on breastfeeding workplace support. The concept of breastfeeding workplace support has economic implications for employers. For this reason, limiting the discussion to the lived experiences of employees clouds our ability to appreciate the differentiated interests at stake and may result in biased policy initiatives. Vilar-Compte and colleagues [14] estimated the cost of maternity leave extension and found that the mean cost of extending maternity leave for 2 weeks in formal employment in Ghana was $109 purchasing power parity. Given the cost associated with maternity leave extension, the perspective of employers on any proposed extension of maternity leave is crucial in balancing the interest of all stakeholders.

### Theoretical framework

Workplace breastfeeding support may facilitate optimal breastfeeding and protect employee mothers from the stress of work and family responsibilities. This assessment of breastfeeding support in healthcare facilities in the Effutu Municipality illuminates further, our understanding of organizational-level factors that impede breastfeeding practice in Ghana. Several theoretical and conceptual frameworks, including the maternal role-incompatibility hypothesis [15], social-ecological theory [12]; work-family conflict framework [16] have been used in studies on work-childcare conflict. This study is predicated on the Social Support Theory (SST). Although the social support theory is mostly used in health and well-being studies [17–19]. This study draws on the stress and coping perspective of the SST as explained by Lakey and Cohen [19] to argue that breastfeeding workplace support can act as stress buffer to support mothers to balance breastfeeding, work stress, and burnout while improving optimal breastfeeding among mothers in paid work.

The social support theory is a broad construct with several definitions [20–23]. It highlights the different forms of aid and assistance provided by family members, friends, neighbors, among others, and often involves a myriad of social interactions [22]. SST is grounded in three different theoretical perspectives, namely, the stress and coping perspective, which states that social support contributes to health by protecting people from the dangers of stress. The second perspective is grounded in social constructionism and proposes a direct relationship between support and health without consideration for stress. The third is the relational perspective, which predicts that the relationship between social support and health cannot be considered without recourse to the relational process that accompanies support [19]. The stress and coping perspective of the SST has principles useful in emphasizing the importance of breastfeeding workplace support to optimal breastfeeding practice.

The values of the modern-day workplace are at variance with those of breastfeeding, often resulting in work-breastfeeding tension, which compromises optimal breastfeeding. Breastfeeding support practices such as a breastfeeding-friendly work environment, maternity leave, breastfeeding breaks, and organizational breastfeeding policy can act as buffers to reduce the stressfulness of mothering and the discrepancies between work and breastfeeding. Providing such support would promote optimal breastfeeding among working mothers. Evidence shows that the provision of breastfeeding rooms, breaks, reduced workloads, and encouragement for breastfeeding mothers promote continuous breastfeeding on return to work [24]. In a community-based cohort study in Taiwan, it was found that mothers with extended maternity leave breastfed longer than those with shorter maternity leave. Lower breastfeeding initiation was also found among mothers returning to work after 1 month [25]. A longitudinal birth cohort study in the US also found that mothers with a longer duration of maternity leave had higher odds of breastfeeding initiation and continuous breastfeeding beyond 6 months [26]. Just as social support contributes to health and wellbeing, These pieces of evidence beacon to us the buffering effect of breastfeeding workplace support in minimizing the negative impact of work-breastfeeding stress on optimal breastfeeding practice.

## Methods

### Study area

The study was rolled out in Effutu Municipality of the Central Region of Ghana. The municipality has a population of 79,411 projected from the 2010 population census and a growth rate of 3.2% per annum [27]. Administratively, Effutu is divided into four sub-municipalities, namely, South-East Winneba, South-West Winneba, Essuekyir-Gyahadze, and Kojo-Bedu North Low-Cost. The municipal health system is organized at the municipal, sub-municipal, and community levels. Each sub-municipality is further divided into communities for purposes of organizing public health services and other health-related services activities. Winneba is the municipal capital and the only urban settlement among the four sub-municipalities.

### Study design and sampling

We used exploratory research design and a mixed-method approach to explore and describe verbal reports of representatives of hospital management and responses of BFHWs on breastfeeding-friendly workplace environment. The study population included all heads of health facilities and bBFHWs in the Effutu Municipality. The selection of the Effutu Municipality proved convenient for two key reasons. First, the municipality was selected based on anecdotal accounts of BFHWs’ challenges of keeping up with the demands of work and mothering. Second, it was selected based on proximity and cost. The lead researcher was introduced to managers of participating health facilities by her affiliated institution. Lists of BFHWs were obtained from participating hospitals. Sampling was done in two stages. In the first stage, four healthcare facilities were purposefully selected based on the number of female employees (20 or more female employees). Selected healthcare facilities included two public healthcare facilities, one private for-profit, and one mission-based healthcare facility. Each facility appointed one representative to respond to our in-depth interviews. The second stage involved a purposeful selection of BFHWs with babies between 3 and 23 months. Mothers in this category were selected because the authors believed they would have fresh lived experiences of work and breastfeeding. In all, a list of sixty-two (62) BFHWs were obtained, and 50 mothers consented to participate in the study.

### Data collection

Both secondary and primary data were used for the study. Secondary data were sourced mainly from reports, handbooks, and published articles. Primary data were gathered through in-depth interviews and self-administered questionnaires with close-ended and Likert-type questions. Questionnaires were used to gather data from BFHWs and in-depth interviews were used to glean data from representatives of hospital management with the aid of an interview guide. The interview guide and questionnaires were both developed by authors based on the literature reviewed [8, 14, 16, 28–30], (see supplementary file 1 for details). After obtaining written consent from the BFHWs, questionnaires were given to participants to complete. Questionnaires were picked up after 1 week, followed by interviews conducted by the lead researcher and assisted by a research assistant. All interviews were conducted in the English language and were recorded using field notes and an audio recorder. Each interview lasted between 40 and 45 minutes. Data for the study were collected between April and May 2018.

### Data analysis

Self-reported questionnaires from BFHWs were processed using Statistical Package for Social Science (SPSS, version 23.0) and analyzed using descriptive statistics. Results on the one hand were presented based on frequencies/percentages and measures of central tendencies. On the other hand, verbal reports of representatives of hospital management were thematically analyzed. Audiotapes were manually transcribed verbatim and validated to guarantee the accuracy of the transcribed data with the audiotapes. Codes were developed manually from the transcribed data to describe their content. Coding was done independently by the first and the second authors and was later compared and reconciled into a single codebook. Common patterns were identified among the codes and selected themes were established based on developed codes. Relevant sentences and phrases were identified and highlighted from the transcript to support the established themes. Saturation was reached when no new themes were identified from the final codes developed by the authors. To ensure that the established themes were representative of the data, the themes were compared against the data set and necessary revisions were made to the initial themes. The themes were redefined to make them useful and coherent.

## Results

### Characteristics of participants

Table 1 presents the characteristics of the study participants. All 50 questionnaires were completed and returned giving a response rate of 100%. Fifty-four (54) respondents participated in the study. Four out of the 54 respondents were representatives of healthcare facilities in the study, were all females, and heads of nursing services. The mean age of participants was 32 years. All participants were females, the majority of whom were married with children between the ages of 4-21 months. The mean age of babies of mothers in the study was 11 months with almost a third (30%) of the babies at 4 months. Sixty-one percent (61%) of BFHWs interviewed were clinical staff and 39% were support staff. Information on parity, work experience, and age in Table 1 relates to BFHWs and babies.

**Table 1:** Demographic characteristics of respondents

Description	Freq.	%	Description	Freq.	%
***Age Respondents***			***parity***		
Mean age	32 ± 5		1	17	34
24–28	14	26	2	13	26
29–33	17	31	3	12	24
34–38	9	17	4	6	12
39–43	9	17	5	2	4
44+	5	9	***Total***	***50***	***100***
Total	***54***	***100***	Work experience		
***Marital Status***			1–4	30	60
Married	43	79	5–8	14	28
Single	8	15	9+	6	12
Divorced	2	3	***Age of Baby (in Months)***		
Widowed	2	3	Mean age of babies	11 ± 5	
***Total***	***54***	***100***	4–6	15	30
***Religion***			7–9	6	12
Christian	32	58	10–12	11	22
Muslim	23	42	13–15	7	14
***Total***	***54***	***100***	16–18	5	10
***Staff Category***			19–21	6	12
Clinical staff	33	61	***Total***	***50***	***100***
Support staff	21	39			
***Total***	***54***	***100***			

Source: constructed by authors using data from the field

Three main themes emerged from the interviews with representatives of health facility management. 1). Standpoints on workplace breastfeeding support. 2). Breastfeeding support. 3). Suggested future directions. Standpoints on workplace breastfeeding support had three sub-themes, namely, Backing for workplace breastfeeding support, Perceived benefits of breastfeeding support, and factors of poor breastfeeding workplace support. Breastfeeding support also had three sub-themes, including maternity protection benefits, workplace support gaps, and awareness creation on benefits. Verbatim quotations were used to support the themes and to provide evidence. Details of the main themes and sub-themes are discussed followed by the quantitative results.

### Standpoints on workplace support for breastfeeding

Representatives of health facility management interviewed shared their facilities’ perceived positions on workplace breastfeeding support and accentuated the importance of providing such support for frontline health workers. The three sub-themes that follow present the details.

### Backings for workplace breastfeeding support

Respondents in this study had extensive knowledge of workplace support for breastfeeding and shared some views on workplace support for breastfeeding. Breastfeeding workplace support such as the creation of breastfeeding rooms where breastfeeding frontline health workers can keep their babies while working was emphasized by all the respondents. The need for a national policy on a breastfeeding-friendly workplace was also mentioned. Respondents believed this would help provide a standardized workplace support culture across industries and workplaces. The representative of hospital 3 shared her views on the need for a breastfeeding-friendly work environment in the following statement:

*Workplaces must have very very well-equipped places for breastfeeding. For instance, in this hospital, there should be a place where working mothers can go and breastfeed. Possibly, there should be a nurse at the place to take care of the babies so that even if the mother is not visiting … and there is bottle feeding, it would be expressed breast milk. It should be a very hygienic place that can take care of babies.*

### The perceived benefits of breastfeeding workplace support

The representative of hospital 4 shared the benefits employers stand to gain when they implement breastfeeding workplace support and said:

*If we have healthy children … . I mean, if a mother can breastfeed so that the child does not have diarrhea and other illnesses, then the mother will not lose working days to go and take care of a sick baby. Once a child is healthy on breast milk, the employee saves money and time for work. So, it is good for mothers in employment and employers as well.*

It is clear from the above statement that creating a breastfeeding-friendly workplace can be thought of as a good business or an aspect of hospitals’ corporate social responsibility. Most respondents were of the view that breastfeeding workplace support has triple benefits that may outweigh the cost involved in providing the support, particularly, loss of man-hours that may arise from the indisposition of infants because of inappropriate feeding practices. The representative of hospital 2 shared this with us.

*Breast milk has nutrients that protect the child from illness, so employers must support it. In this way, working mothers will not need to take days off work to care for their babies because of illness. Mothers will always be present at work to discharge their duties. I believe employers stand to benefit if their employees are always present at work.*

### Factors of poor breastfeeding workplace support

In this study, a range of factors was outlined by respondents to limit efforts in providing breastfeeding support for BFHWs. They included a lack of funding to create a breastfeeding-friendly workplace environment, limited office space, and inadequate staff. Two of the respondents retorted that it is difficult to promote breastfeeding supportive work culture, particularly when more frontline health workers must be provided with such support at the same time. The representative of hospital 2 shared her experience and said:

*Hmm … it is quite difficult. Sometimes we have issues with limited staff. Supposing you are on a ward that requires ten (10) staff but only eight (8) are on duty and you have some of the staff taking two (2) hours off to breastfeed. … . this situation puts pressure on the other remaining staff if the workplace is busy. They are forced to do the work of those breastfeeding in addition to theirs, especially when the breastfeeding mothers decide to take some time off their schedules to breastfeed. Other times too, you may have situations where more than three mothers would be on maternity leave at the same time in a unit. This gives more work and stress to the other staff who may be at work.*

### Breastfeeding support

All health facilities in the study had some form of breastfeeding support in place for BFHWs. Predominantly, they are those guaranteed in the maternity protection provision of the Labor Act of Ghana. The details are discussed in the sub-themes below.

### Maternity Protection benefits

As part of the conditions of service of BFHWs, breastfeeding mothers are granted 12 weeks paid maternity leave in the case of spontaneous vagina delivery (SVD) and 24 weeks for those with assisted delivery, such as cesarean section. BFHWs also enjoy paid breastfeeding breaks. Respondents from both private and public hospitals mentioned that the conditions of service of BFHWs capture all the maternity protection provisions provided in the labor laws of Ghana. Further, BFHWs are first put on morning shifts only until the baby is 26 weeks, followed by morning and afternoon shifts until the baby is 52 weeks old. They can also apply for casual leave where necessary.

### Workplace support gaps

The gaps in breastfeeding workplace support identified in the health facilities are summed up in the statements of the representatives of hospital 1 and 3 as follows:

*Our clients breastfeed in the wards … …… … . I mean our postnatal inpatients are entitled to breastfeed in the ward. For our staff, we do not have a lactating site. A breastfeeding staff must leave her baby at home and when it is time to breastfeed, she goes home to breastfeed. If they are fortunate to have a babysitter who will come to work with them, then they can bring their babies along to breastfeed at work … … maybe sit under the tree if they would be comfortable over there. But as to getting a place that is so conducive for breastfeeding, no, not at all.* (Representative of hospital 1).

*When they come to work, depending on the workload for the day … you know, there are days that the workplace is very busy and there are days that the workplace is less busy. On our busy days, when we monitor and realize that the tension has reduced, we give them the time to go and breastfeed. Sometimes, when the workplace is busy, they communicate with their babysitters and ask, ‘is the baby in need of breast milk?’, ‘is it time for breastfeeding?’ ‘is baby showing any signs of lactating?’ If it comes out like that then the person goes and then breastfeeds.* (Representative of hospital 3).

These statements highlights the plight of BFHWs and the work-breastfeeding discrepancies that are likely to result from it. Almost all hospitals in the study did not have breastfeeding facilities or an on-site creche for kids of BFHWs. Only one out of the four hospitals was constructing an onsite creche at the time of the study.

### Awareness creation on breastfeeding workplace support

Even though healthcare facilities in the study did not have most of the essentials of a breastfeeding-friendly workplace environment, those provided in the condition of services of BFHWs, such as maternity leave, casual leave, working half-day, and other staff welfare packages were disseminated among BFHWs through staff orientation, memos, website, and staff meetings. It was also assumed that BFHWs are well informed of issues related to optimal breastfeeding practice, given that breastfeeding education is integrated into maternity services.

### Suggested future direction

Respondents were united in their views regarding the way forward. They called for a revision of the Labor Act, Act 651 of 2003 to define the essentials of the workplace breastfeeding policy. On the flipside, representatives of the mission-based and private hospitals expressed a preference for a policy guideline that would leave details of action for promoting a breastfeeding-friendly workplace environment to employers to formulate and implement based on organization-specific circumstances. The representative of hospital 4 explained further in the statement below:

*A national policy would be helpful. Yes, it would guide the hospitals to formulate their action plans. I think a policy framework must come from the top and then translated down to all healthcare facilities. Individual hospitals can have their strategic plans which will have considerations for specific needs related to breastfeeding mothers.*

To obtain balanced views on the extent of support for workplace breastfeeding, BFHWs were interviewed. Table 2 presents BFHWs’ views of breastfeeding workplace support and breastfeeding practice. Participants’ views on breastfeeding workplace support were consistent with responses from the interviews. Ninty-6 % (96%) of the participants said they went on 12 weeks maternity leave, had breastfeeding break (80%), and 70% indicated that they enjoyed five-day 20-h workweek (5/20) (i.e., half-day) after returning to work instead of the usual five-day 40-h workweek (5/40). Participants mentioned staff orientation (44%) and workshops (16%) as the main sources of maternity protection benefits information.

**Table 2:** Mothers’ Views on Workplace Support

	***Freq.***	***%***			
***Breastfeeding policy at workplace***			***Mothers’ feeding strategies***		***%***
Yes	0	0	Expressed breast milk	17	34
No.	48	96	Breastfeed when at home	20	40
No response	2	4	Breast milk and porridge	13	26
***Paid maternity leave***			***Feeding trategy below 6 months***		
Yes	48	96	Expressed breast milk	18	36
No.	2	4	Artificial milk	2	4
***Paid breastfeeding Break***			Breast milk and water	9	18
Yes	40	80	porridge, water, and breast milk	6	12
No.	10	20	No response	15	30
***Worked half-bay***			***Had breastfeeding education when pregnant***		
Yes	35	70	Yes	47	94
No	5	10	No	3	6
No response	10	20	***Aware of breastfeeding benefits***		
***Sources of maternity benefits information***			Yes	50	100
HR handbook	3	6	No.	0	0
Facility’s website	3	6	***Initiated breastfeeding***		
Orientation	22	44	Yes	50	100
Workshop	8	16	No	0	0
Memos/circulars	4	8	***Reporting/closing time affected breastfeeding***		
No. response	10	20	Yes	20	40
***Policy allows oing to work withbBaby***			No	30	60
Yes	45	90	***Would sacrifice work to breastfeed***		
No	5	10	Yes	48	96
***Go to Work long with baby***			No	2	4
Yes	7	14	***The possible effect of separation on breastfeeding***		
No.	43	86	Insufficient breast milk	***8***	***16***
***Breastfeeding facility at workplace***			Early winning	17	34
Yes	3	6	Decision not to breastfeed	4	8
No.	47	96	No response	21	42
***Reasons for leaving baby at home***			***Common breastfeeding challenges***		
To concentrate at work	8	16	Work overload	19	38
No place to keep baby	14	28	Insufficient breastmilk	11	22
wanted the baby to be at creche	2	4	Breast milk contamination	10	20
No response	26	52	Breast milk expression	10	20
Mean reporting time to work	8:00 am		***Common coping strategies***		
Mean closing time from work	3:00 pm		Husband/relatives support	35	70
			Colleagues’ support	9	18
			Closing at unapproved times	7	12

Source: constructed by authors using field data

The results also established several shortfalls in the breastfeeding workplace support of the hospitals. Ninety-six percent (96%) of breastfeeding frontline health workers indicated a lack of breastfeeding policy and breastfeeding facilities (94%) in their hospitals. Even though 90% said their workplace policy allows them to go to work along with baby, only 14% go to work with their babies, possibly due to the lack of breastfeeding facilities or creche in the hospitals. However, reasons offered for not going to work with baby were, no place to keep the baby (28%) and concentration at work (16%). A little above half of the participants failed to provide reasons. All (100%) BFHWs in the study knew the benefits of breastfeeding and did initiate breastfeeding. However, about 34% of them supplemented breastfeeding with artificial milk (4%), water (18%), and porridge and water (12%). Averagely, participants indicated that they reported to work at 8:00 am and closed at 3:00 pm each day. Feeding strategies upon returning to work included breast milk expression (34%), breastfeeding when at home (40%), and breastmilk and porridge (26%). Twenty percent of the participants indicated that their closing and reporting time affected breastfeeding and 96% said they will prioritize work over breastfeeding in circumstances of work-breastfeeding conflict. About a third (34%) of the respondents indicated they would wean their babies earlier than planned due to the separation between them and their babies occasioned by the demands of work.

Table 3 presents mothers’ evaluation of coping strategies adopted to manage breastfeeding-work tension. The results show social support such as support from husbands and relatives (median = 3) as highly supportive in mitigating the inconsistencies between work and childcare. Flexible work arrangement (median = 2) and support from coworkers (median = 2) were believed to be moderately supportive for coping with the challenges arising from work, breastfeeding, and childcare. Regarding the challenges of work and breastfeeding, undue stress resulting from the conflict between work and breastfeeding (median = 3) was considered extremely challenging. Insufficient breast milk arising from the separation between mother and baby (median = 2), difficulties in expressing milk (median = 2), work overload (median = 2), and work duration (median = 2) were considered moderately challenging.

**Table 3:** Mothers’ Evaluation of Coping Strategies of Childcare, Work, and Challenges

Description	Frequency					
***Challenges***	Least Challenging (1)	Most Challenging (2)	Highly Challenging (3)	NR	Total	Median
Difficulties in expressing milk	12	17	5	16	50	2.0
Inadequate breast milk due to infrequent feeding	9	16	10	15	50	2.0
Breastmilk contamination	14	10	9	17	50	2.0
Work overload	7	15	21	7	50	2.0
Work duration	12	15	9	14	50	2.0
Conflicting responsibilities	11	12	15	12	50	2.0
Stress and burnout	5	15	21	9	50	3.0
Poor concentration at work	6	14	17	13	50	2.0
Difficulties in meeting timelines	10	12	11	17	50	2.0
***Copping Strategies***	Least Supportive (1)	Moderately Support (2)	Highly Supportive (3)	No Response	Total	Median
Avoiding workplace responsibilities	18	13	2	17	50	1.0
Reporting to work late	15	10	4	21	50	1.0
Leaving workplace before approved closing time	17	8	4	21	50	1.0
Support from husband and relatives	3	7	34	6	50	3.0
Flexible work arrangement	6	22	9	13	50	2.0
Breast milk expression	7	10	19	14	50	3.0
Support from colleagues	2	18	11	19	50	2.0

Source: constructed by authors using field data

## Discussion

In this study, we assessed the extent to which health facilities in the Effutu Municipality provide a breastfeeding-friendly workplace environment for BFHWs, their views on the breastfeeding support they receive, and the coping strategies used in managing work-breastfeeding challenges. Out of the list of 62 BFHWs obtained, only 50 consented to participate in the study. Consequently, the views expressed in this study are the views of BFHWs who actually participated in the study. Even though the study recorded a 100% response rate, it must be noted that some of the questionnaire items recorded non-response possibly because participants had limited time to complete the questionnaires. The results are likely to have some amount of nonresponse bias. The results established that hospital leaders have a positive standpoint on breastfeeding workplace support that has practical value. However, effort at implementing the standpoints appeared restrained by systemic challenges. BFHWs who go to work along with their babies had no access to demarcated places for babies. The challenge with this situation is that babies do not have a fully developed immune system, exposing them to the hospital environment has the potential to compromise their health. Breastfeeding sites and onsite creches in hospitals could be a haven for preventing infants and kids of BFHWs from hospital-acquired infections. Other gaps included a lack of education on strategies for returning to work and balancing childcare and work.

The findings show that the most reliable workplace breastfeeding support available to BFHWs for breastfeeding/childcare were maternity leave, spousal, and co-worker support. Even though family support was prominent, it is at best appropriate for childcare compared to breastfeeding. Family support exists outside of the workplace and may not represent adequate social support for improving optimal breastfeeding on return to work. It also demonstrates a lack of workplace support systems for breastfeeding mothers to cope with the stress and burnout arising from work and breastfeeding in the health facilities studied. Systems at the workplace to promote optimal breastfeeding among mothers returning to work are an important aspect of the International Labor Organization’s (ILO) recommendation for maternity protection benefits [29]. Women have the right to work, and children have the right to appropriate feeding and nutrition. It is therefore important that pragmatic steps are taken to promote breastfeeding workplace environment in Ghana. Even though the conditions of service of BFHWs subscribe to the maternity protection benefits enshrined in the Labor Act, the provisions are not enough to support and protect optimal breastfeeding on return to work.

A notable gap identified was the lack of breastfeeding policy in the hospitals. This circumstance can markedly affect breastfeeding upon return to work. Though some institutions in Ghana have systems in place to support women to balance the demands of work, learning, and childcare, Ghana has no policy framework that guarantees a minimum package of workplace breastfeeding/childcare support for working mothers. In the US for instance, the Patient Protection and Affordable Care Act (ACA) oblige employers with 50 or more employees to allow reasonable break time and provide designated rooms for breast milk expression [30, 31]. Even though there is a dearth of empirical literature on the effectiveness of national policies on workplace breastfeeding protection in improving breastfeeding initiation and duration [32], Kogan et al. [33], have found a relationship between state adopted breastfeeding support laws and breastfeeding initiation and duration in developed economies. Likewise, Kim et al. [34], conducted a systematic review on the effectiveness of workplace lactation interventions on breastfeeding in the US and found a significant association between interventions such as the provision of a breast pump, return-to-work consultation, telephone support, and exclusive breastfeeding duration. While we do not seek to create economic and institutional equivalence between Ghana and the US, the above evidence shows that effective national policies that enforce provisions of basic lactation support in the workplace, particularly, in health facilities would help create employee value and make breastfeeding-friendly accredited hospitals worth their names.

Health facility representatives interviewed blamed their inability to provide such an environment on lack of resources. They believe that if their hospitals were to have adequate resources and implementation guidelines, they could undertake such initiatives for staff. It stands to reason that employers are unlikely to provide breastfeeding support at the workplace in the absence of a policy directive that obliges them to do so. On this basis, a national policy in this regard can help promote a breastfeeding workplace culture in healthcare institutions. The views of respondents on the triple benefits of providing breastfeeding workplace support corroborates the argument that breastfeeding workplace support is cost-effective compared to the extension of maternity leave. It reduces absenteeism, improves employee retention, and increases employee morale and loyalty [35–37].

Workplace breastfeeding support has mutual benefits to both employers and employees. On this basis, partnership between industry and policymakers can be explored for improving workplace breastfeeding in Ghana. Such a partnership can yield positive outcomes for both employers and employees. Available evidence indicates that workplace lactation interventions are related to better outcomes such as lower absenteeism, lower turnover, higher job satisfaction, and positive organizational reputation [38, 39]. The absence of such interventions may lead to poor work outcomes. The situation in Ghana puts employers in a disadvantaged position in the absence of such a partnership. Breastfeeding mothers would accept to work given the financial benefit they stand to earn, coupled with the lack of jobs that guarantee job security. Yet, breastfeeding mothers may lack the level of morale and job satisfaction needed for improved productivity.

Poor breastfeeding workplace environment in this study may well be the bane of optimal breastfeeding practice as BFHWs find it an enormous task to continue breastfeeding upon return to work [40]. Smith and colleagues in their study found a significant association between awareness of breastfeeding support policy and higher rates of exclusive breastfeeding among Australian working mothers. Supportive work culture such as a flexible work arrangement for breastfeeding mothers varied across hospitals in this study. The arrangements were unofficial and were granted based on exigencies at the workplace and at the discretion of unit heads and managers. Such an arrangement is unlikely to support exclusive breastfeeding. Evidence from Durban, USA, shows that women in workplaces that had supportive work culture exclusively breastfed 4 months and longer than those in workplaces with poor supportive work culture [40].

The results of this study and the literature in Ghana seem to suggest that breastfeeding break and maternity leave are the common forms of breastfeeding support culture of organizations in Ghana.

BFHWs’ access to maternity leave and breastfeeding break corroborate research findings from the USA and Ghana [13, 41]. Due to the lack of a breastfeeding facility and the desire to concentrate at work, most BFHWs breastfeed when they are at home. This practice was also found in some universities in Nigeria where most breastfeeding employee-mothers breastfeed before and after work only [42].

Almost all participants indicated receiving breastfeeding education during pregnancy, which presupposes that BFHWs are aware of the benefits of exclusive breastfeeding. Yet, 34% supplemented breastfeeding with water, artificial milk, and porridge on return to work, when the baby was below 6 months. This situation could be attributed to the negative effect of maternal work on breastfeeding and accentuates the need for intervention in the workplace to minimize the effect of work-related factors on optimal breastfeeding. The findings of this study also emphasize frontline health workers’ desire for economic independence and how this quest can influence infant feeding practice. Most women are eager to work in return for economic benefit. As evident in this study, 70% of the participants indicated that they would not sacrifice work for breastfeeding in situations where breastfeeding interferes needlessly with work, which is consistent with earlier research in Ghana [15]. In this research, it was found that maternal work affected breastfeeding in situations where income from maternal work was needed to supplement the family budget. It is even possible to assume that net income of BFHWs who participated in this study formed a significant proportion of their family’s budget.

Averagely, the participants spent 6–7 h in the workplace, and others (40%) believed that time spent in the workplace affected breastfeeding. This makes BFHWs the most affected in the absence of workplace breastfeeding policy. They have a dual duty to fulfill their gendered role of infant nursing and work to support their families. Breastfeeding and childcare support at the workplace are aspects of employee welfare. Under this circumstance, the Ghana Registered Nurses and Midwives Association, the Health Service workers Union and the Ghana Medical Association can play a vital role in pressing home the urgent need for breastfeeding support policy in hospitals. Support from husbands and relatives in this study is consistent with research findings in Ghana, where family support aided exclusive breastfeeding practice among working mothers [8].

The proposed future directions provided by respondents point to the need for extensive stakeholder consultation in arriving at a national workplace breastfeeding and childcare support policy. For instance, there were divergent views on the extension of maternity leave from 3 to 6 months further to WHO’s push for an increase in maternity leave. While the Ghana Medical Association, Nutritionists, and some mothers were in favor of such a policy, other women in paid employment were more interested in the institutionalization of baby-friendly workplace environments to enable them to return to work for fear of losing their jobs [5]. The opinions shared by stakeholders in the literature and in this study provide deeper insights into what should be the content of future policy on workplace breastfeeding policy. Most importantly, the process for such a policy should entail extensive stakeholder engagement to ensure that workplace support policies provide equitable benefits to employers and employees.

### Limitations of the study

The results of this study have limited generalizability owing to the use of non-probability sampling and descriptive data analysis. It is also possible that the background of respondents interviewed might have introduced some level of bias in the verbal responses. Given that respondents were females and members of management of their respective hospitals. The data collected only allowed for the description of mothers’ views on workplace breastfeeding support and did not capture mothers’ lived experiences of navigating the difficult terrine of modern employment and child nursing. Similarly, the study did not explore workplace lactation interventions and their significance for BFHWs’ work output and optimal breastfeeding. This can be considered for further studies.

## Conclusion

We conclude based on the analysis of the interviews and questionnaire responses that breastfeeding support provided to BFHWs in the Effutu Municipality is limited to those enshrined in the Labor Act. Health facilities in the study do not provide a breastfeeding-friendly work environment for BFHWs. The BFHI has no significance for the workplace environment of staff in the frontline of implementation of the BFHI. Consistent with the social support theory, support from relatives was perceived as the most effective buffer against work-breastfeeding stress and tension. Continuous advocacy on breastfeeding workplace support and stakeholder engagement to build consensus on the mix of strategies appropriate to cushion BFHWs for optimal breastfeeding and improved productivity is recommended. Sensitization of employers on the benefit of workplace lactation interventions to improved productivity is also recommended.

## Supplementary Information

**Additional file 1.** Research instrument file. Questionnaire and interview guide. The file contains the questionnaire and interview guide used to glean data for the study.

## Data Availability

The datasets used and/or analyzed during the current study are available from the corresponding author on reasonable request.

## Abbreviations

WHO: World Health Organization
BFHI: Baby-Friendly Hospital Initiative
SST: Social Support Theory
