# Supplementary material for: Towards a comprehensive breastfeeding-friendly workplace environment: insight from selected healthcare facilities in the central region of Ghana
Source: BMC Public Health. 2021 Sep 9;21:1647. doi: 10.1186/s12889-021-11652-5 (PMC8427943; doi:10.1186/s12889-021-11652-5)
Supplement: Supplementary file 1 — Additional file 1. Research instrument file. Questionnaire and interview guide. The file contains the questionnaire and interview guide used to glean data for the study. [file 12889_2021_11652_MOESM1_ESM.docx]

**QUESTIONNAIRE – WORKPLACE BREASTFEEDING SUPPORT**

Hello! We are researching workplace support for breastfeeding among breastfeeding front-line health workers in the Effutu municipality. Our questionnaire is divided into six main sections (A - F). We would be grateful if you could provide us with the requested information by answering the questions under each of the six sections. We assure you that all information you would provide in this questionnaire would be treated as confidential and would be used purely for academic purposes or publication.

**SECTION A**

**Socio-demographic information**

Please, answer the following questions by checking (√) as appropriate.

1. Age [ ]
2. Marital Status: Married [ ]; Single [ ]; Divorced [ ]; Widowed [ ]; Others Specify…………….
3. Religion: Christianity [ ]; Muslim [ ]; Others ……………………
4. Number of children ……………………
5. Age of baby being breastfed……………
6. How long have you been working in this organization?.......................
7. Facility type: Public [ ]; Private [ ].

**SECTION B:**

**Breastfeeding Practices**

Please, answer the following questions by checking (√) as appropriate and write the requested information in the blank spaces provided.

1. Did you receive education on exclusive breastfeeding when pregnant ? Yes [ ]. No. [ ].
2. Which of the following places did you receive breastfeeding education?

Antenatal clinic [ ], labor ward [ ], postnatal ward [ ], child welfare clinic

[ ]. Other (specify)……………………………………………………………

1. Are you aware of the benefits of breastfeeding to your child’s health? Yes [ ]. No. [ ].
2. If yes, which of the following are you aware of? Has appropriate nutrients in right proportions for baby [ ], it provides the right nourishment for baby [ ], it has the right quantity of water that baby needs [ ], it protects baby from diarrhea [ ], Other (specify)……………………………………..………….
3. Did you initiate breastfeeding. Yes [ ]. No [ ].
4. Which of the following breastfeeding type did you practice in the first six months? Please, check as appropriate. Breast milk only for baby [ ], Breast milk, water and or other liquids [ ], Breast milk, porridge and or baby milk
5. [ ]. Other (specify)……………………………………………………………

**SECTION C**

**Breastfeeding Policy**

Please, answer the following questions by checking (√) as appropriate and write the requested information in the blank spaces provided.

1. Do you have an explicit policy on breastfeeding in your workplace? Yes [ ].

No [ ].

1. If yes, have you ever read the policy? Yes [ ]. No [ ].
2. Which of the following facilities do you have in the workplace? Please, check (√) as appropriate. Breastfeeding breaks [ ], Breastfeeding facilities [ ], Promotion of work-based childcare [ ], breastfeeding package for expectant mothers [ ], flexible work arrangement [ ], flexible leave arrangement [ ]. Other (specify)…………………………………………………………………….
3. Which of the following are provided in your workplace?
4. Paid maternity leave Yes [ ]. No [ ].
5. Paid breastfeeding break? Yes [ ]. No [ ].
6. ‘half day’ Work? Yes [ ]. No [ ].
7. Is the extension of maternity leave allowed in the case of cesarean section or related complications? Yes [ ]. No [ ],
8. Which of the following are allowed in addition to maternity leave? Please, check (√) as appropriate. Flexible work arrangements [ ] Paid breastfeeding breaks

[ ], Flexible leave arrangements [ ]. [ ], Leave without pay [ ], Casual leave [ ].

1. Which of the following sources do you receive information on breastfeeding and childcare? HR handbook [ ], facility’s website [ ], during orientation [ ], workshops and training by HR [ ], memos and circulars [ ]. Other (specify)

……………………………

**SECTION D**

**Breastfeeding Facilities**

Please, answer the following questions by checking (√) as appropriate and write the requested information in the blank spaces provided.

1. Does your workplace policy allows you to go to work along with baby? Yes [ ].

No [ ].

1. Do you go to work along with the baby? Yes [ ]. No [ ].
2. If you go to work along with the baby, do you go with a nanny? Yes [ ]. No. [].
3. How often do you breastfeed the baby in the workplace? Breastfeed on demand [ ], breastfeed when I feel like it [ ] when am a bit free [ ], anytime baby cries [ ]. Other (specify)………………………………
4. Do you have a place dedicated to breastfeeding and the expression of breastmilk in the workplace? Yes [ ]. No [ ].
5. If no, where do you sit to breastfeed? Office [ ], kitchenette [ ], office porch

[ ], washroom [ ], unused office [ ], other specify……………………………

1. If yes, what type of facility is it? Please, check (√) as appropriate. On-site nursery or crèche [ ], Lactation room (i.e., a room for breastfeeding) [ ]. Other (specify)………………………….

**SECTION E**

**Challenges of Breastfeeding and Work**

Please, answer the following questions by checking (√) as appropriate and write the requested information in the blank spaces provided.

1. If you do not go to work along with the baby, which of the following reasons account for that? Have a nanny/relative in the house to care for baby [ ], want to concentrate at work [ ], do not have a place to keep baby [ ], wants the baby to be at the crèche [ ]. Other (specify)…………………………………
2. If you do not go to work along with the baby, how do you breastfeed? Expressed breast milk [ ], breastfeed when at home [ ]. Breast milk and porridge [ ]. Other (specify)…………………………
3. Which of the following feeding practices did you adopt when the baby was below six months? Expressed breast milk [ ], artificial milk [ ], water and breast milk [ ], water porridge, and breast milk [ ]. Others (specify)…………………
4. Which of the following does the practice of not going to work along with baby create? Insufficient breast milk [ ], early weaning of baby [ ], Decision not to breastfeed [ ]. Others (specify)……………………………………………
5. Which of the following would you consider as a challenge to breastfeeding? Please, check (√) as appropriate.
6. Breast milk expression [ ].
7. Insufficient milk resulting from infrequent breastfeeding [ ].
8. Contaminated milk resulting from poor storage [ ].
9. Work overload [ ].
10. Close late often times [ ].
11. At what time do you usually report to work? ………………………………
12. At what time do you usually close to work? .............................................
13. Does reporting and closing time affect breastfeeding? Yes [ ], No[ ]
14. If yes, how does it affect breastfeeding? Insufficient milk [ ], early weaning

[ ], early introduction of complementary foods [ ], the decision not to breastfeed [ ].

1. Will you consider sacrificing work to enable you to breastfeed?

Yes [ ]. No [ ].

1. On a scale of 1 – 3, how would you rate the work and breastfeeding challenges in the table? Please, check (√) as appropriate.

| Breastfeeding challenges | 1  Least challenging | 2  Moderately challenging | 3  Highly challenging |
| --- | --- | --- | --- |
| Difficulty in expressing breast milk |  |  |  |
| Inadequate milk resulting from infrequent breastfeeding |  |  |  |
| Milk contamination as a result of poor storage |  |  |  |
| Work overload |  |  |  |
| Work duration |  |  |  |
| Conflicting Responsabilités |  |  |  |
| Stress and burnout |  |  |  |
| Poor Concentration at work |  |  |  |
| Have difficulties meeting deadlines |  |  |  |

1. Do you consider these challenges important to the baby weaning decision?

Yes [ ]. No. [ ].

**SECTION F**

**Mechanisms for Coping with Breastfeeding Challenges**

Please, answer the following questions by checking (√) as appropriate.

1. Which of the following mechanisms do you employ to cope with breastfeeding, childcare, and work?
2. Avoiding some work responsibilities [ ].
3. Report to work late [ ].
4. Leave work place before approved closing time [ ].
5. Support from husband and relatives [ ].
6. Flexible work scheduling [ ].
7. Support from colleagues [ ].
8. Leave without pay [ ].
9. On a scale of 1 – 3, how would you rate the coping mechanisms you have selected above in the table below? Please, check (√) as appropriate.

| Coping strategies | 1  Least supportive | 2  Moderately supportive | 3  Highly supportive |
| --- | --- | --- | --- |
| Avoide some work responsabilités |  |  |  |
| Reporting to work late |  |  |  |
| Leave the Workplace before approved closing time |  |  |  |
| Support from husband and relatives |  |  |  |
| Flexible work scheduling |  |  |  |
| Use of expressed milk |  |  |  |
| Support from colleagues |  |  |  |

1. Do you consider breastfeeding/childcare support from the organization as an important intervention for promoting breastfeeding and childcare?

Yes [ ] No [ ].

1. If yes, what do you think your organization must do to help you manage to breastfeed and work effectively?………………………………………

……………………………………………………………………………….

**INTERVIEW GUIDE**

**WORKPLACE BREASTFEEDING FRIENDLY ENVIRONMENT**

Dear Sir/Madam,

We are researching workplace breastfeeding-friendly environments of health facilities in the Effutu municipality. We would be happy if you could spend some time with us and respond to some questions on the topic. Our conversation would be recorded analyzed later for publication. However, we promise to anonymize your responses and also keep them safe by research ethics.

**Employers' Knowledge of Breastfeeding and Breastfeeding Friendly Work Environment.**

Do you consider breastfeeding as an important aspect of childcare?

1. Why do you think breastfeeding is important?
2. What are your views on breastfeeding workplace support?
3. In your views, what do you think should be the elements of breastfeeding workplace support?

**Breastfeeding Policy**

1. As a hospital, do you have an explicit policy on breastfeeding in the workplace?
2. How do you ensure that front-line health workers breastfeed as recommended by WHO/UNICEF?
3. How do you ensure that front-line health workers are aware of the maternity protection benefit and other staff welfare policies?
4. How often do you communicate the privileges to them?

**Maternity Protection Benefits**

1. Which of the following benefits do you guarantee for expectant mothers and what are the details?
2. Maternity leave
3. Other types of leave and conditions
4. Breastfeeding break
5. Other work schedule arrangements for breastfeeding front-line health workers.

**Challenges of Ensuring Breastfeeding Workplace Environment**

1. What exactly accounts for the shortfall in the childcare and breastfeeding related support you provide for front-line health workers?
2. What do you intend to do differently to promote and support breastfeeding in the workplace?
3. What do you think should be the way forward to protect and promote optimal breastfeeding among front-line health workers?

**Breastfeeding Facilities**

1. What are the views of the leadership of this hospital on providing breastfeeding facilities for front-line health workers?
2. Do you have such facilities in this hospital?
3. If no, how are breastfeeding front-line health workers able to breastfeed optimally?
4. If you have, are all your staff aware of this privilege?

**Breastfeeding Program**

As a healthcare facility, do you have a breastfeeding program for front-line health workers?

What is the content of the program, and how do you go run it?
